# The Hydrophobic Residues in Amino Terminal Domains of Cx46 and Cx50 Are Important for Their Gap Junction Channel Ion Permeation and Gating

**DOI:** 10.3390/ijms231911605

**Published:** 2022-10-01

**Authors:** Roa’a Jaradat, Xiaole Li, Honghong Chen, Peter B. Stathopulos, Donglin Bai

**Affiliations:** Department of Physiology and Pharmacology, University of Western Ontario, London, ON N6A 5C1, Canada

**Keywords:** gap junction structure, Cx46, Cx50, amino terminal domain, V_j_-gating, single channel conductance, patch clamp

## Abstract

Lens gap junctions (GJs) formed by Cx46 and Cx50 are important to keep lens transparency. Functional studies on Cx46 and Cx50 GJs showed that the V_j_-gating, single channel conductance (γ_j_), gating polarity, and/or channel open stability could be modified by the charged residues in the amino terminal (NT) domain. The role of hydrophobic residues in the NT on GJ properties is not clear. Crystal and cryo-EM GJ structures have been resolved, but the NT domain structure has either not been resolved or has showed very different orientations depending on the component connexins and possibly other experimental conditions, making it difficult to understand the structural basis of the NT in V_j_-gating and γ_j_. Here, we generated missense variants in Cx46 and Cx50 NT domains and studied their properties by recombinant expression and dual whole-cell patch clamp experiments on connexin-deficient N2A cells. The NT variants (Cx46 L10I, N13E, A14V, Q15N, and Cx50 I10L, E13N, V14A, N15Q) were all able to form functional GJs with similar coupling%, except Cx46 N13E, which showed a significantly reduced coupling%. The GJs of Cx46 N13E, A14V and Cx50 E13N, N15Q showed a reduced coupling conductance. V_j_-gating of all the variant GJs were similar to the corresponding wild-type GJs except Cx46 L10I. The γ_j_ of Cx46 N13E, A14V, Cx50 E13N, and N15Q GJs was reduced to 51%, 82%, 87%, and 74%, respectively, as compared to their wild-type γ_j_s. Structural models of Cx46 L10I and A14V predicted steric clashes between these residues and the TM2 residues, which might be partially responsible for our observed changes in GJ properties. To verify the importance of hydrophobic interactions, we generated a variant, Cx50 S89T, which also shows a steric clash and failed to form a functional GJ. Our experimental results and structure models indicate that hydrophobic interactions between the NT and TM2 domain are important for their V_j_-gating, γ_j_, and channel open stability in these and possibly other GJs.

## 1. Introduction

Gap junctions (GJs) promote direct intercellular communications by permitting the passage of not only ions (Na^+^, K^+^, Ca^+2^, Cl^−^), but also metabolic (glucose, amino acid) and signaling molecules (cAMP, cGMP, inositol trisphosphate (IP3)) that are under 1 kDa in size, thereby optimizing tissue homeostasis and normal organ function [1,2,3]. Consequently, dysfunction in GJs has been shown to play a role in a variety of pathological conditions such as stroke, cardiac arrythmia, deafness, cataract, skin disorders, and several types of cancers [4,5,6,7]. GJs are composed of different integral protein subunits, named connexins. Six connexins oligomerize into a cylindrical channel shape across the plasma membrane and form a connexon or hemichannel. Two hemichannels dock together to form one gap junction channel [1,8]. There are 21 connexin genes in the human genome. Different connexins vary in their protein size and amino acid sequence yet all connexins share a similar topological structure with four transmembrane domains (TM1–TM4) connected by two extracellular loops (E1 and E2) and one cytoplasmic loop (CL), as well as the cytosolic amino and carboxyl termini (NT and CT, respectively) [9]. Transmembrane domains, extracellular loops, and the NT domain are highly conserved among different connexins [10]. Different tissues express one or more connexins, which can form different gap junction channels that serve specialized tissue-specific functions [9].

Functional characterization of GJs formed by different connexins shows diverse channel properties, including a wide range of ion permeation rates, distinct selectivity to different ions and small molecules, and different gating control by divalent cations (e.g., Ca^2+^ and Mg^2+^), pH levels, and transjunctional voltage (V_j_). Transjunctional voltage-dependent gating (also known as V_j_-gating) is a common property of all GJs. GJs formed by different connexins display distinct levels of V_j_-gating extent, half deactivation voltage, gating sensitivity (also known as gating charge), and/or gating kinetics [11,12]. The rate of ion permeation (measured by single channel conductance or γ_j_) of GJs formed by different connexins range from a few pico-Siemens (pS) to hundreds of pS [13,14,15,16]. The molecular and structural mechanisms for controlling GJ channel gating properties and ion permeation are not fully clear.

Many electrophysiological studies provide compelling evidence that the amino-terminal domain (NT) and the residues within the NT of Cx26, Cx32, Cx40, Cx46, and Cx50 are critical for their V_j_-gating polarity, gating extent, gating sensitivity and kinetics, channel open stability, and single channel conductance (γ_j_) [17,18,19,20,21,22,23,24,25,26,27,28]. Most of these studies have been more focused on the charge changes in the NT region or the entire NT domain and support a GJ-gating model where the NT domain exists in the pore and is capable of sensing the V_j_, also contributing, at least partially, to the electrostatic properties of the pore for ion permeation. Other residues, including hydrophobic residues, have not been clearly defined with respect to their role in V_j_-gating and ion permeation.

The first high-resolution (3.5 Å) crystal structure resolved was of human Cx26 GJ where the NT domain folded into the channel and lined the vestibule part of the GJ pore. Collectively, six NT domains of each hemichannel form a funnel structure comprising the narrowest part of the GJ [29,30]. These structural features of the NT domain make it suitable for sensing voltage differences across the channel and suggest that it forms a constriction site to regulate the rate of ion permeation and selectivity, which could affect both V_j_-gating and single channel properties. Molecular simulation studies based on this structure model have revealed that the NT domain of Cx26 is highly dynamic and the structure of the NT could be rapidly disordered without chemical modification or freezing [31,32,33]. Consistent with these studies, a few GJ structure models of Cx26 were unable to resolve the NT domain structure [34,35], questioning the structural anchoring mechanism for the NT domain in the GJ pore. More recently, high-resolution cryogenic electron microscopy (Cryo-EM) structures of native sheep Cx46 and Cx50 GJs, at 3.4 Å and later 1.9 Å resolutions, were resolved [18,36]. Most domains in Cx46 and Cx50 show a similar structure as those of Cx26, including the NT domains in Cx46 and Cx50 GJs, which are positioned in the vestibule to form the narrowest part of the channel. However, this NT domain displayed the largest structural difference compared to Cx26 with very different orientations and local interactions. Specifically, the NT domain adopts an amphipathic α-helix in which the hydrophobic amino acids are packed against the transmembrane (TM1 and TM2) domains forming hydrophobic interactions, which stabilize the NT domain in the open conformation of Cx46 and Cx50 GJs while the hydrophilic side is facing into the pore, potentially playing a role in voltage sensing and ion/substrate selectivity [18,36]. Hydrophobic interactions between NT and TM1/2 domains in Cx46 and Cx50 GJ are a novel mechanism explaining how the NT may be anchored and stabilized in the pore. In addition, some NT residues at positions 9–15 are not perfectly resolved in the density map [36], indicating that they either show very different conformations in Cx46 compared to Cx50 or that these residues are very dynamic with multiple possible orientations, establishing different intra- and/or inter-subunit hydrophobic and/or salt-bridge interactions. We compared the conformation of the NT domain between Cx46 and Cx50 after structural alignment of the remaining polypeptide chain and found that Cx46 Phe6 and His17 adopt very different orientations compared to Cx50 (Figure S1), indicating different side chain packings to accommodate the residue differences within the NT domains. These NT structural differences and distinct single channel conductances as well as open dwell times in these two connexins indicate a role for non-conserved (i.e., non-identical) NT residues in determining the unique channel properties of these GJs. Here, we tease out the effects of each of these residue types individually. It is interesting to note that hydrophobic residues in the NT domains of all connexins are highly conserved [37], underscoring their critical role in GJ function.

**Figure 1:**
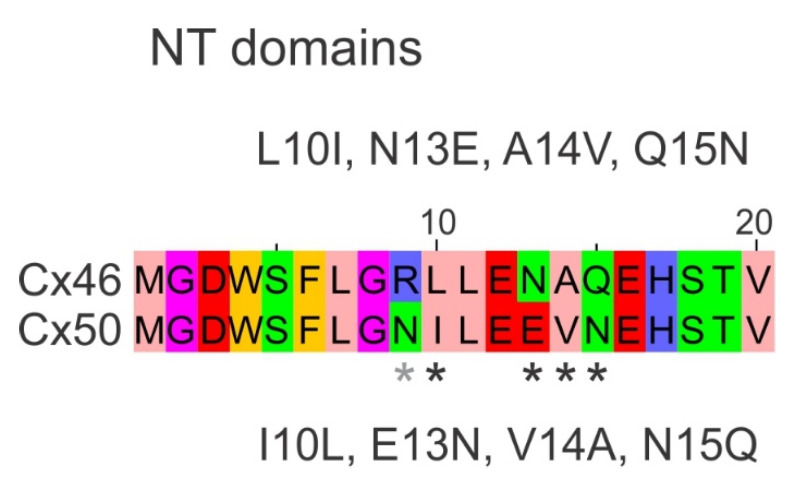
Amino acid sequence alignment of Cx46 and Cx50 amino terminal domains. The first 20 amino acid residues of the amino terminal (NT) domains of Cx46 and Cx50 are aligned. A total of 5 out of 20 residues are different as indicated by asterisks (black and grey). Our designed missense variants are listed next to their parental connexin. A grey asterisk on the ninth position indicates that both Cx46 N9R and Cx50 R9N were studied earlier [38].

To start testing these novel structural features and their influences on V_j_-gating and ion permeation in the Cx46 and Cx50 connexins, we designed a series of missense variant pairs in the sheep Cx46 and Cx50 NT domain where we switched non-identical residues one by one as shown in Figure 1 and studied the V_j_-gating properties and single channel conductance of these variants with the dual patch clamp technique. Our experimental data together with homology modeling in this and our previous study [38] demonstrated an important role for hydrophobic interactions between the NT and TM1/2 domain in GJ channel V_j_-gating and unitary channel conductance in these lens GJs and possibly other connexin GJs.

## 2. Results

### 2.1. Homotypic Cx46 and Cx50 Variants Form Functional GJ Channels

**Figure 2:**
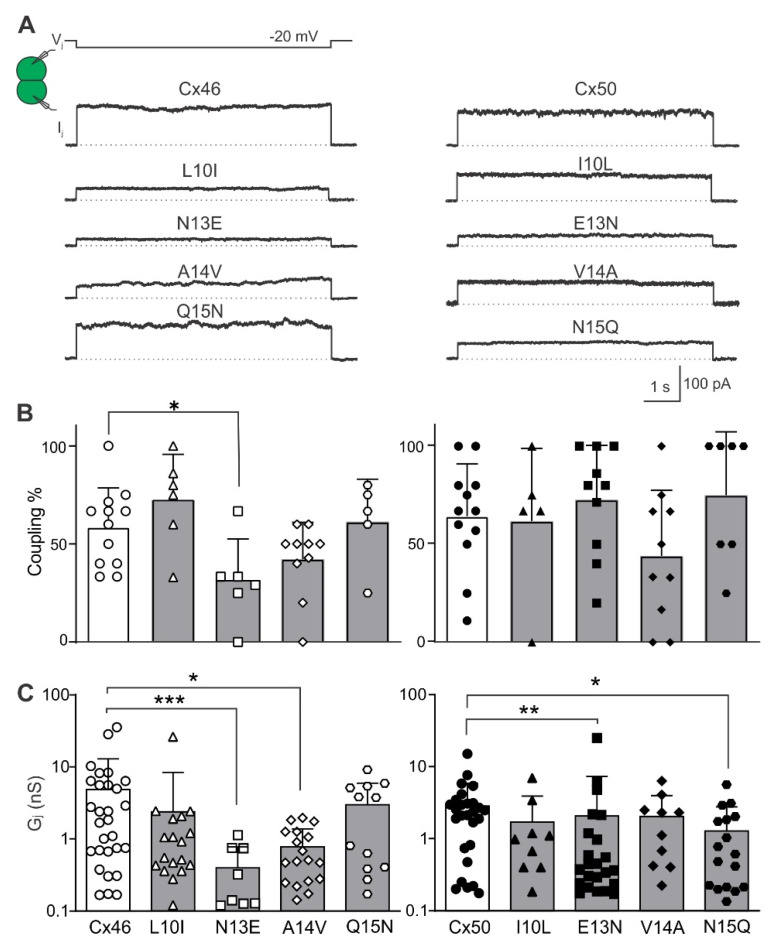
Coupling percentage and conductance (G_j_) of N2A cell pairs expressing homotypic wild-type Cx46, Cx50, and their variants on 10th, 13th, 14th, and 15th positions in the NT domain as indicated. (**A**) A typical transjunctional current (I_j_) is shown for N2A cell pairs expressing one of the designed missense variants or wild-type as indicated. Expression of each of these variants successfully led to GJ coupling with I_j_s recorded in response to a −20 mV V_j_ pulse delivered to the other cell of the pair as shown. (**B**) Bar graphs illustrate mean coupling% + SD for each of the Cx46 variants (left panel) or Cx50 variants (right panel). Each data point represents coupling% for one transfection and 3–10 successful recordings were included to calculate coupling% for each transfection. Only the Cx46 N13E variant showed a reduced coupling% compared to Cx46 (* *p* = 0.014) with Kruskal–Wallis test followed by Dunn’s test. (**C**) Bar graph shows averaged coupling conductance (G_j_) of coupled N2A cell pairs expressing each of the variants as indicated. The error bars represent SD. Cell pairs expressing Cx46 N13E and A14V showed significantly lower G_j_ compared to Cx46, while Cx50 E13N and N15Q displayed a significantly reduced G_j_ compared to Cx50. Kruskal–Wallis test followed by Dunn’s test was used to compare each variant G_j_ to the corresponding wild-type G_j_. Empty vector-transfected (IRES-GFP) N2A cells were used as negative controls for these experiments and none of the negative control cell pairs showed any I_j_s. Statistical significance is indicated with asterisks (* *p* < 0.05; ** *p* < 0.01, *** *p* < 0.001). Different shaped symbols in the bar graph indicate individual measurements of the corresponding variants shown at the bottom.

To investigate the effects of switching individual NT residues between the Cx46 and Cx50 on their ability to form functional GJs, we generated four pairs of point variants where a single residue at the 10th, 13th, 14th, or 15th positions of the NT domain was switched with the equivalent residue between Cx46 and Cx50 (see Figure 1). These point variants are L10I, N13E, A14V or Q15N in Cx46, and I10L, E13N, V14A or N15Q in Cx50. All of these variants, as well as wild-type Cx46 and Cx50, were inserted in an expression vector with unfused GFP as a reporter, for example Cx46 L10I-IRES-GFP. Transient transfection of each of these constructs into N2A cells resulted in successful expression of GFP. Isolated GFP-positive cell pairs were selected for dual patch clamp to check their coupling status. GJ coupled cell pairs were readily identifiable with successful recording of junctional currents (I_j_s) in response to a 20 mV V_j_ pulse (Figure 2A). Cell pairs expressing wild-type Cx46 showed an average coupling percentage (or coupling%) of 59 ± 20%, and for Cx50 the coupling% was 64 ± 27% (Figure 2B). Recordings were also made on cell pairs expressing each of our designed variants and the coupling% of all the variants were not statistically different from their corresponding wild-type connexin, except for Cx46 N13E where a reduced coupling% (31 ± 21%, * *p* = 0.014) was observed, indicating that all these variants, except N13E, were able to form functional GJs similar to their wild-type connexins. We also studied localization of GFP tagged Cx46 N13E using HeLa cells and found that Cx46 N13E-GFP could be found at the cell–cell interface to form GJ plaque-like structures (Figure S2), similar to that of wild-type Cx46-GFP, indicating that the GJ functional impairment of this variant is unlikely due to an inability to reach the plasma membrane.

Next, coupling conductance (G_j_) was calculated from coupled cell pairs expressing one of the variants or wild-type connexin (Figure 2C). The average G_j_s of Cx46 L10I (2.38 ± 6.01 nS, n = 18) and Q15N (2.98 ± 2.97 nS, n = 12) were similar to that of wild-type Cx46 (4.85 ± 8.09 nS, n = 29, *p* > 0.05 for each of these two variants). In contrast, the G_j_s of Cx46 N13E (0.39 ± 0.38 nS, n = 9, *** *p* < 0.001) and A14V (0.77 ± 0.60 nS, n = 18, * *p* = 0.0104) were significantly reduced compared to wild-type Cx46 (Figure 2C). The G_j_s of the Cx50 I10L (1.70 ± 2.20 nS, n = 9) and V14A (2.03 ± 1.94 nS, n = 10) variants were similar to wild-type Cx50 (2.84 ± 3.07 nS, n = 27, *p* > 0.05 for each of these two variants), whereas the G_j_s of Cx50 E13N (2.07 ± 5.24 nS, n = 23, ** *p* =0.004) and N15Q (1.28 ± 1.48 nS, n = 17, * *p* = 0.024) were significantly reduced compared to wild-type Cx50 (Figure 2C).

### 2.2. V_j_-Gating Properties of the Homotypic GJs Formed by Cx46 or Cx50 NT Variants

**Figure 3:**
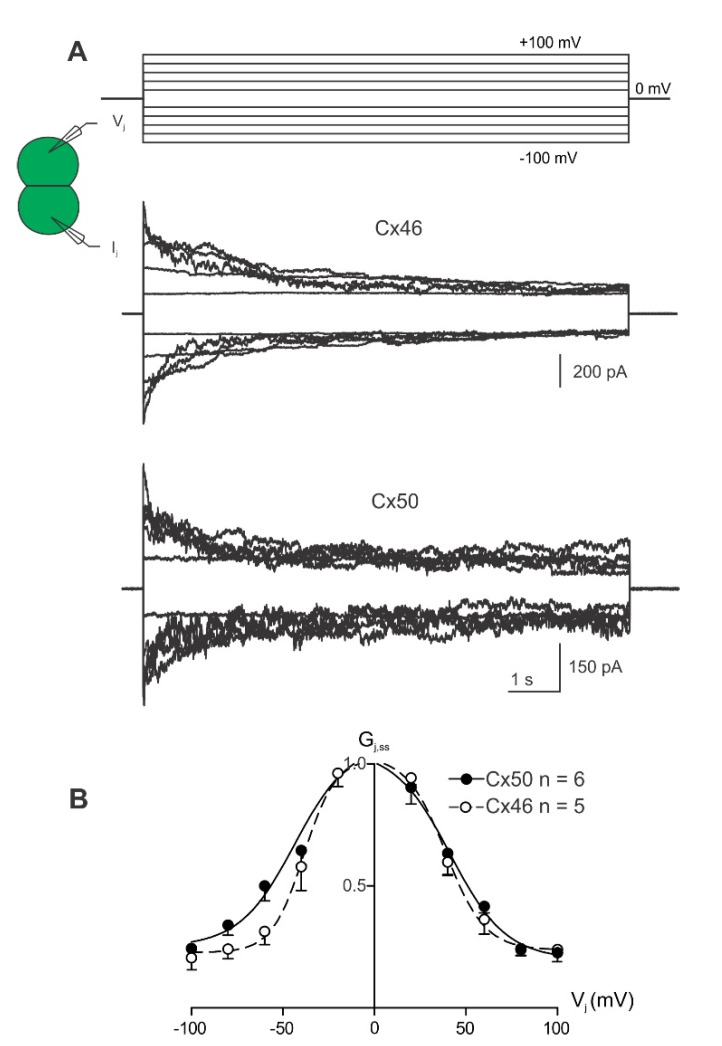
V_j_-gating of homotypic Cx46 and Cx50 gap junction channels. (**A**) Superimposed junctional currents (I_j_s) from cell pairs expressing wild-type Cx46 or Cx50 in response to a series of voltage pulses (V_j_s) ranging from ±20 to ±100 mV in 20 mV increments as shown at the top. (**B**) Normalized steady-state junctional conductance (G_j,ss_) was plotted against the tested V_j_ range for Cx46 (open circles) and Cx50 (solid circles) GJs. A Boltzmann equation was used to fit the G_j,ss_–V_j_ plot of Cx46 (smooth dashed grey lines) and Cx50 (smooth solid grey lines). Error bars represent SD. Number of cell pairs analyzed are indicated.

To investigate transjunctional voltage-dependent gating (V_j_-gating) of cell pairs expressing one of the Cx46 or Cx50 NT variants, dual whole-cell voltage clamp technique was used with one of the cells stepping to a series of voltage pulses (±20 to ±100 mV in 20 mV increments) and recording junctional current (I_j_s) from the other cell. The I_j_s from cell pairs expressing either Cx46 or Cx50 showed V_j_-dependent mirror-symmetrical deactivation in response to those absolute V_j_s ≥ 40 mV (Figure 3A). The steady-state conductance near the end of each I_j_ is normalized to the peak conductance to obtain the normalized steady-state conductance (G_j,ss_), which was plotted as a function of tested V_j_s (G_j,ss_–V_j_ plot as shown in Figure 3B). Data in the G_j,ss_–V_j_ plots for both Cx46 and Cx50 GJ could be fitted well with Boltzmann equations on each V_j_ polarity to obtain the following parameters: G_max_, the maximum normalized conductance; G_min_, the minimum normalized conductance; half deactivation voltage V_0_ (at which the normalized conductance = (G_max_ + G_min_)/2); and the slope *A*, which describes the V_j_-gating sensitivity. The G_j,ss_–V_j_ plot revealed near mirror-symmetrical G_j,ss_ reduction for both V_j_ polarities (Figure 3B). The Boltzmann equation fit both datasets well, with the extracted G_min_, G_max_, V_0_ and *A* parameters not significantly different between the wild-type Cx46- and Cx50-expressing cell pairs.

**Figure 4:**
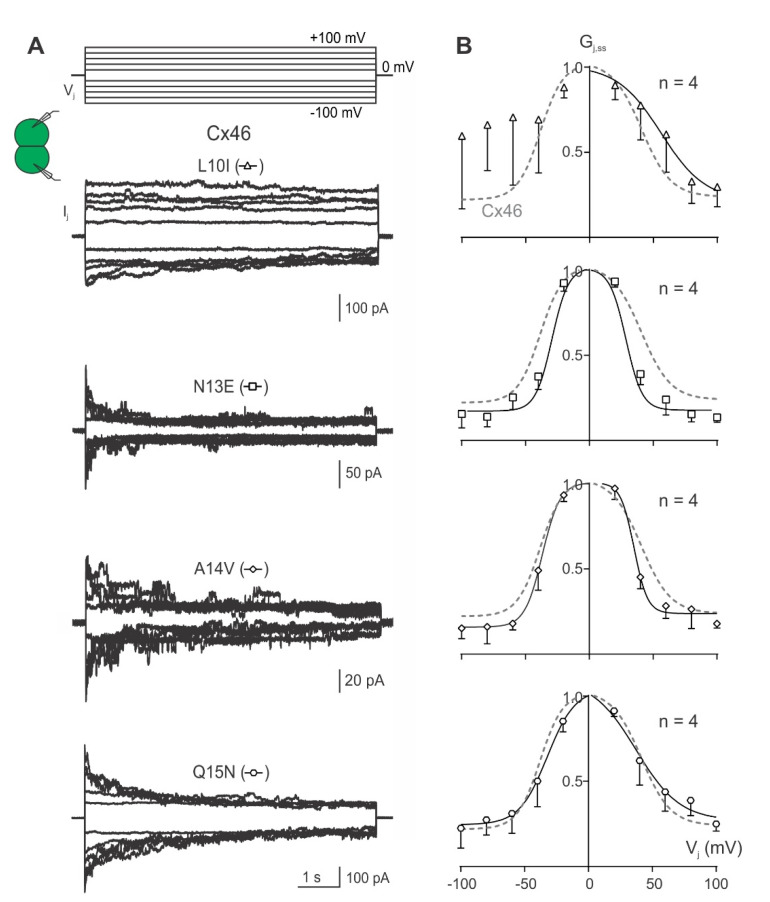
V_j_-gating properties of GJs formed by Cx46 variants, L10I, N13E, A14V, and Q15N. (**A**) Superimposed I_j_s from cell pairs expressing L10I, N13E, A14V, and Q15N are illustrated in response to a series of voltage pulses (V_j_s) ranging from ±20 to ±100 mV in 20 mV increments as shown at the top. (**B**) Normalized steady-state junctional conductance (G_j,ss_) plotted against tested V_j_ range for L10I (open triangles), N13E (open squares), A14V (open diamonds), and Q15N (open hexagons) GJs. The Boltzmann fitted curves (smooth solid black lines) for each of the variants are superimposed with those of wild-type Cx46 (smooth dashed grey line) for easy direct comparison. The number of cell pairs for each variant is indicated on each plot. Error bars represent SD.

Next, I_j_s were recorded followed by the assessment of G_j,ss_–V_j_ plots for each of the Cx46 NT variants: L10I, N13E, A14V, and Q15N. Cell pairs expressing the N13E, A14V or Q15N variants showed near-symmetrical deactivation of I_j_s for both V_j_ polarities, whereas cell pairs expressing L10I showed I_j_ deactivation on one of the V_j_ polarities but inconsistent I_j_ deactivation on the other V_j_ polarity (Figure 4A). All variant-derived G_j,ss_–V_j_ plots could be fit well with Boltzmann equations, except the −V_j_ for L10I, where no consistent V_j_-gating was observed in one of the V_j_ polarities (Figure 4B). The Boltzmann fitting parameters from the GJs formed by the Cx46 NT variants were not significantly different from those of wild-type Cx46 GJ (Table 1).

**Figure 5:**
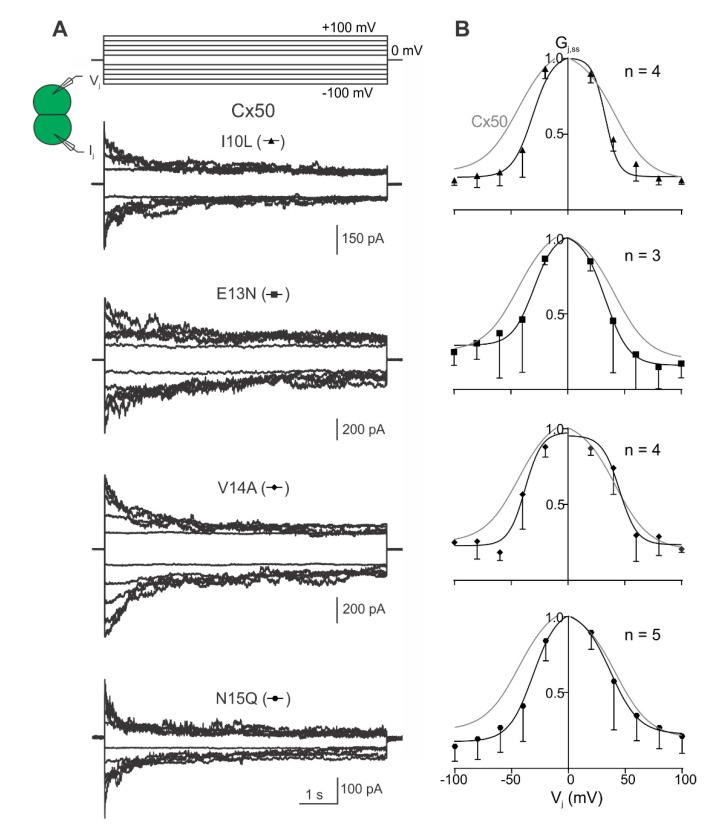
V_j_-gating properties of the GJs formed by Cx50 variants, I10L, E13N, V14A, or N15Q. (**A**) Superimposed junctional currents (I_j_s) from cell pairs expressing I10L, E13N, V14A, and N15Q are illustrated in response to a series of voltage pulses (V_j_s) ranging from ±20 to ±100 mV in 20 mV increments. (**B**) Normalized steady-state junctional conductance (G_j,ss_) was plotted against a tested V_j_ range for I10L (solid triangles), E13N (solid squares), V14A (solid diamonds), and N15Q (solid hexagons) GJs. The Boltzmann fitted curves (smooth solid black lines) for variants and for wild-type Cx50 (smooth solid grey lines) are superimposed for direct comparison. The number of cell pairs for each variant is indicated on the respective plot. Error bars represent SD.

Similarly, I_j_s were recorded in response to the V_j_ protocol in cell pairs expressing each of the Cx50 NT variants: I10L, E13N, V14A, or N15Q. The GJs formed from each of these variants showed near symmetrical I_j_s which deactivated to a similar level at V_j_s ≥ 40 mV (Figure 5A). Each of the Cx50 NT variant-derived G_j,ss_–V_j_ plots was fitted well with a Boltzmann equation for each V_j_ polarity (Figure 5B). The Boltzmann fitting parameters of these Cx50 NT variants were not significantly different from those of wild-type Cx50 at each corresponding V_j_ polarity (Table 2).

### 2.3. Unitary Channel Conductance of Homotypic GJs of Cx46 and Cx50 NT Variants

**Figure 6:**
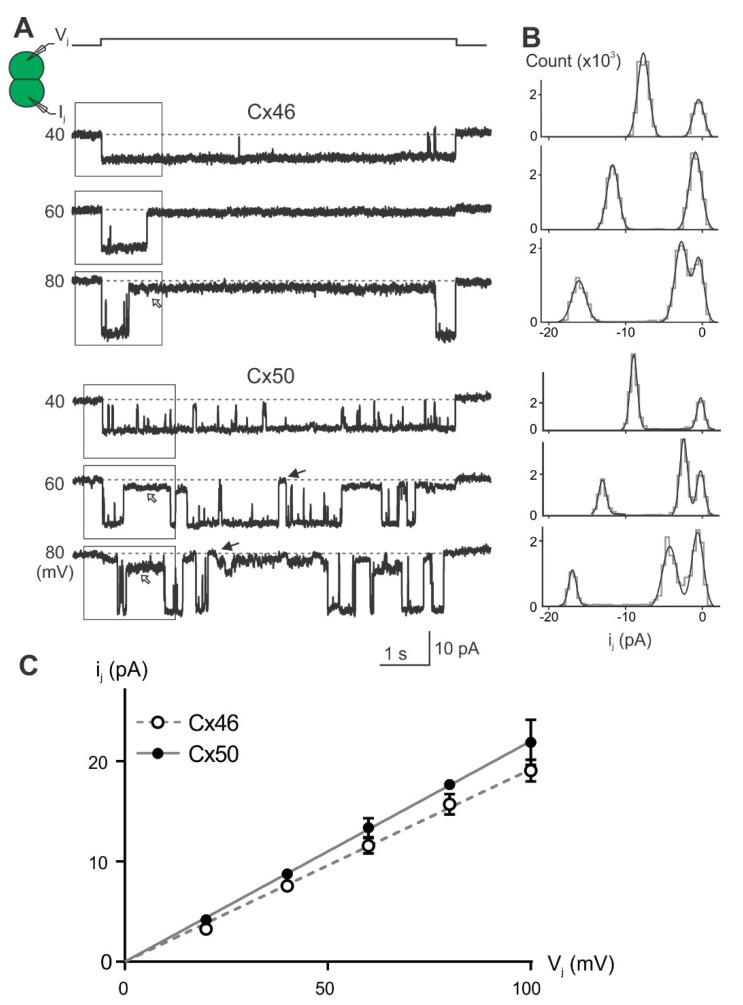
Unitary channel properties for wild-type Cx46 and Cx50 GJs. (**A**) Single channel currents (i_j_s) recorded as a response to the administration of positive V_j_ pulses (+V_j_) as indicated. After the channels are fully open, they enter either a subconductance state (open arrows) or a fully closed state (solid arrows). Boxed areas represent the region used to create the corresponding all-point histograms shown on the right for each V_j_. (**B**) All-point histogram (grey lines) and Gaussian fits (smooth black lines) for i_j_s of Cx46 and Cx50 at 40, 60, and 80 mV V_j_ pulses, showing the i_j_ amplitude at the main open state and fully closed state. Subconductance states were also identified at some V_j_s. (**C**) Scatter plot of i_j_s amplitude at the main open state for the entire tested range of V_j_s. Linear regression of each i_j_–V_j_ plot was used to obtain the slope, which represents slope unitary channel conductance (γ_j_) for Cx46 or Cx50.

To study how each Cx46 or Cx50 NT point variant affected the rate of ion permeation in individual GJ channels, we recorded unitary channel currents (i_j_s) in poorly coupled cell pairs at different V_j_s. Typical i_j_s from cell pairs expressing wild-type Cx46 or Cx50 showed increasing amplitudes as a function of increasing V_j_s (Figure 6A). The first ~2 s of the i_j_s (boxed areas outlined at the beginning of i_j_s) were used to generate an all-point histogram (Figure 6B grey lines), which was fitted by two or more Gaussian functions (Figure 6B smooth black lines) to measure i_j_. The i_j_ amplitudes at the main open state, fully closed state, and in some cases subconductance states could be identified (Figure 6B). The i_j_ amplitudes of the main open state were plotted at the tested V_j_s (Figure 6C), and linear regression of the i_j_–V_j_ plot was used to obtain the slope unitary channel conductance (γ_j_). The average slope γ_j_ of Cx46 GJ is 192 ± 5 pS (n = 5), whereas the average slope γ_j_ of Cx50 GJ is 220 ± 7 pS (n = 5, ** *p*= 0.006).

**Figure 7:**
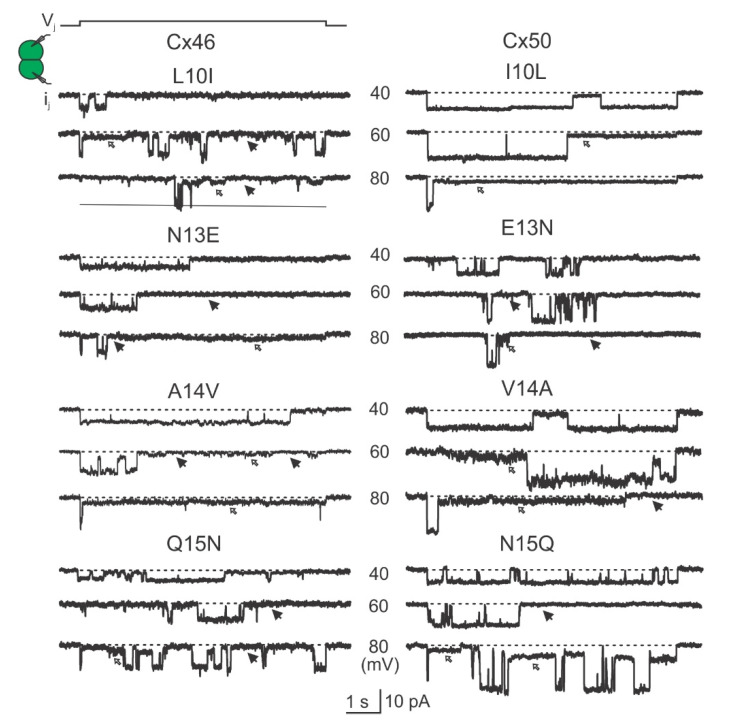
Single channel currents (i_j_s) recorded in response to the indicated V_j_s. Representative i_j_s are shown for the homotypic GJs formed by Cx46 NT variants (left panels) or Cx50 NT variants (right panels). In addition to the fully open state, subconductance (open arrows) and fully closed (solid arrows) states were also observed for some i_j_s.

**Figure 8:**
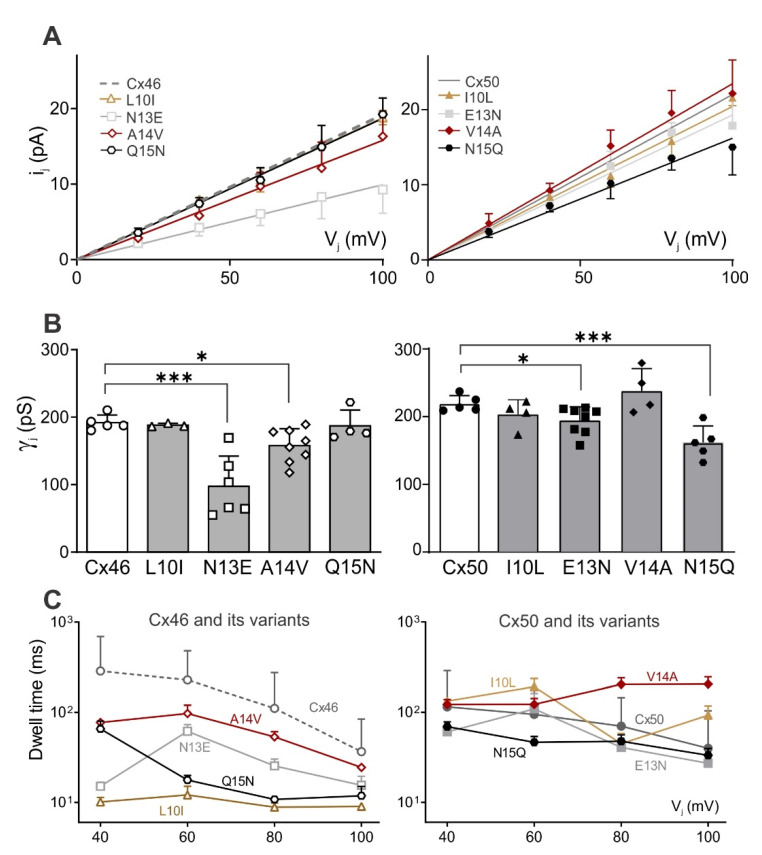
Unitary channel properties of homotypic GJs of Cx46 L10I, N13E, A14V, Q15N and Cx50 I10L, E13N, V14A, N15Q variants. (**A**) Plot of averaged i_j_ amplitudes at the main open state for the tested V_j_s. Linear regression was used to obtain the slope which represents the slope unitary channel conductance (γ_j_) for each variant. The dashed and solid gray lines represent the slope γ_j_s of Cx46 and Cx50, respectively. The slope γ_j_ of Cx46 L10I is 188 ± 6 pS (n = 3), N13E γ_j_ is 93 ± 17 pS (n = 6), A14V γ_j_ is 94 ± 11 pS (n = 6), Q15N γ_j_ is 187 ± 10 pS (n = 4), Cx50 I10L γ_j_ is 204 ± 10 pS (n = 4), E13N γ_j_ is 193 ± 17 pS (n = 8), V14A γ_j_ is 234 ± 12 pS (n = 4), and N15Q γ_j_ is 162 ± 14 (n = 5). (**B**) Bar graphs for direct comparison of slope γ_j_s of tested Cx46 (left panel) and Cx50 (right panel) variants. Cx46 N13E and A14V as well as Cx50 E13N and N15Q showed significantly lower γ_j_ compared to their corresponding wild-type. Kruskal–Wallis test followed by Dunn’s test was used to compare each variant to its corresponding wild-type. (**C**) Average open dwell time for each GJ was plotted at different V_j_s. The number of events analyzed ranged from 25 to 637 from two to nine cell pairs. Note that the open dwell time is plotted on a logarithmic scale to show large differences among these GJs. * *p* < 0.05; *** *p* < 0.001.

Representative unitary channel currents (i_j_s) of all the NT point variants at the 10th, 13th, 14th, and 15th positions of Cx46 (left panel) and Cx50 (right panel) are shown in Figure 7. In most cases, the i_j_s showed higher open probability (P_open_) for the main open state at V_j_ of 40 mV than those at higher absolute V_j_s (60 or 80 mV). During the larger V_j_ pulses (60 or 80 mV), both residual state (open arrows) and fully closed state (solid arrows) were observed (Figure 7). All-point histograms and Gaussian function fits were used to measure the amplitudes of i_j_s at the main open state for each tested V_j_. The linear regression of an i_j_–V_j_ plot for each of the Cx46 variants was used to obtain the slopes γ_j_ and these were plotted together with the wild-type Cx46 slope γ_j_ (Figure 8A left panel). The slope γ_j_ of Cx46 N13E and A14V were significantly decreased compared to the slope γ_j_ from wild-type Cx46 (93 ± 17 pS for N13E, *** *p* < 0.001 and 158 ± 25 pS for A14V, * *p* = 0.022). Similarly, the i_j_–V_j_ plot for each of the Cx50 variants was generated and linear regression was used to obtain the slope γ_j_s. The wild-type Cx50 slope γ_j_ was used as a direct comparison to the variants (Figure 8A right panel). The slope γ_j_s of Cx50 E13N and N15Q GJs were significantly decreased compared to the wild-type Cx50 GJ (193 ± 17 pS for E13N, * *p* = 0.026; and 162 ± 14 pS for N15Q, *** *p* < 0.001).

As shown in Figure 6 and Figure 7, the open dwell time for Cx46, Cx50, and their variant GJs appeared to be very different. To quantify the open dwell time for each of these GJs, the i_j_s of these GJs were further analyzed to obtain an open dwell time for each open event at the tested V_j_s and were subsequently averaged and plotted as a function of tested V_j_s (Figure 8C). It is clear that the Cx46 GJs showed the longest dwell time at different V_j_s. All of the designed Cx46 NT variant GJs showed a significantly shorter dwell time at all tested V_j_s (Figure 8C, Table S1), indicating the open state stability is decreased in these variants. In contrast, Cx50 GJs showed a similar level of dwell time with those of its variants, except V14A at the higher V_j_s (80 and 100 mV) or N15Q at the lower V_j_s (40–80 mV, Figure 8C, Table S1), indicating that there is no consistent change in the open state stability for these tested Cx50 NT variants.

### 2.4. Homology Modeling of Selected Cx46 and Cx50 Variants

**Figure 9:**
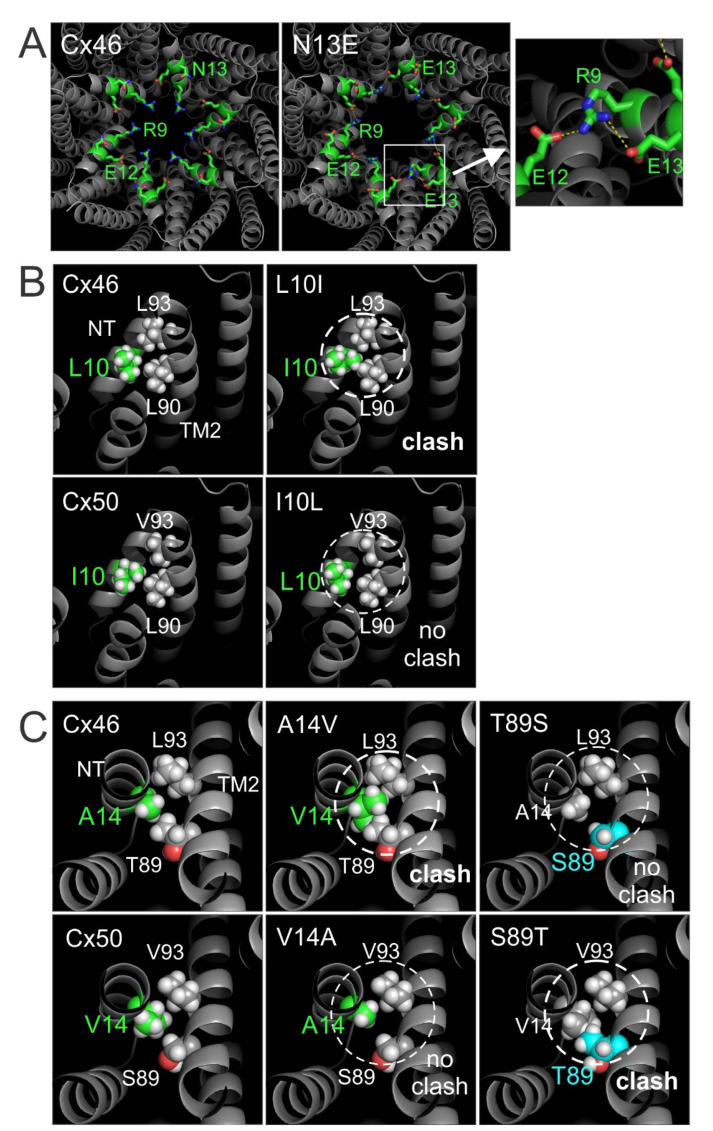
Structural models for selected variant GJs. (**A**) Cx46 GJ model determined by Cryo-EM (PDB: 7JKC) is shown on the left panel with a viewing angle facing the pore entrance. Homology model of Cx46 N13E is shown in the middle panel to show that R9 and E12 frequently interact with one another via salt-bridges. On one occasion, the side chain of E13 also interacts with the side chain of R9 of the same subunit to form a salt-bridge interaction (see enlarged view on the right panel. Note that R9 also formed a salt-bridge interaction with E12 of a neighboring subunit as shown). (**B**) Zoomed view of Cx46 and Cx50 highlighting the interactions of the 10th residue with 90th and 93rd residues on the TM2 domain (left panels). Cx46 L10I caused a steric clash between I10 and L90, while the Cx50 I10L did not lead to any steric clash with L90 or V93 (right panels). (**C**) Zoomed view of Cx46 and Cx50 highlighting the interactions of the 14th residue with 89th and 93rd residues on the TM2 domain (left panels). Cx46 A14V caused a steric clash between V14 and T89 or L93, while Cx50 V14A did not lead to any steric clash at the equivalent residues (middle panels). Similarly, Cx50 S89T caused a steric clash with V14, while no clash was observed with Cx46 T89S (right panels).

To begin exploring the structural and mechanistic insights of our experimental results, homology models were developed for several variants showing the most interesting changes in GJ properties. For example, Cx46 N13E was selected as its GJs showed significantly lower coupling%, reduced G_j_, shorter open state dwell time, and a large reduction in γ_j_. The homology model of Cx46 N13E showed that E13 could form an intra-subunit salt-bridge interaction with R9 as shown in Figure 9A. The probability of this interaction as shown in Figure 9A is not very high (only one out of six E13 residues interacts with R9); however, we believe that this interaction pulls R9 toward the cytosol direction, which could destabilize the NT from the open conformation to promote GJ closure or a reduced permeation passage. This structural effect may have partially contributed to our observed reduction in coupling%, open state dwell time, G_j_, and γ_j_. We cannot rule out other possibilities at this time.

Cx46 L10I is the only variant that caused large changes in GJ V_j_-gating. A simple exchange of this residue on the Cx46 structure model (7JKC) resulted in a steric clash between I10 and L90 on the TM2 domain (Figure 9B), suggesting that these residues could play a role in maintaining normal V_j_-gating properties, while the mirroring variant, Cx50 I10L, on Cx50 GJ structure (7JJP) did not show any steric clash (Figure 9B) and their V_j_-gating properties appeared to be similar to the wild-type Cx50 GJ (see Figure 5).

Another interesting pair of variations occurs on hydrophobic residues, i.e., Cx46 A14V and Cx50 V14A. In wild-type Cx46 and Cx50, the 14th residue packs tightly with two residues (89th and 93rd) in the TM2 domain through hydrophobic interactions, as shown in Figure 9C. When valine was introduced in Cx46 (A14V) through simple exchange, the larger side chain of valine produced a steric clash with L93 and T89 (Figure 9C), which is likely to destabilize the NT domain, possibly pushing it into the GJ pore to reduce the pore size and leading to a reduced G_j_ and γ_j_. The mirror variant, Cx50 V14A, displayed no steric clash (Figure 9C), and the NT domain may pack closer to the TM2 due to the smaller side chain, widening the GJ pore diameter. Nevertheless, we observed no experimental evidence for such widening of the GJ pore, as we did not observe any increase in the single channel conductance and the G_j_ of Cx50 V14A was also similar to that of Cx50.

In addition to simple exchange of the above two hydrophobic residues on experimentally resolved structures, we also developed homology structure models of Cx46 L10I, A14V and Cx50 I10L, V14A to assess how the full set of NT residues may reorient to accommodate each of these substitutions. As shown in Figure S3, the NT residues showed various degrees of changes; moreover, residues in other domains, especially at TM2, also showed changes to accommodate these variants.

To further study the importance of the hydrophobic interactions between the A14/V14 and 89th or 93rd residues in the TM2 domain, we also modeled variants at the 89th position in Cx46 and Cx50. Simply mutating the 89th residue on the Cx46 or Cx50 GJ models predicted that Cx50 S89T causes a steric clash with V14 of the same subunit (Figure 9C), while Cx46 T89S did not lead to any steric clash as A14 and S89 are both relatively small side chains. Consistently, our homology structure models of these two variants also indicated that Cx50 S89T causes larger structural changes in the NT domain residues than that of Cx46 T89S (Figure S4), indicating that more reorienting of these NT residues is required to accommodate Cx50 S89T.

**Figure 10:**
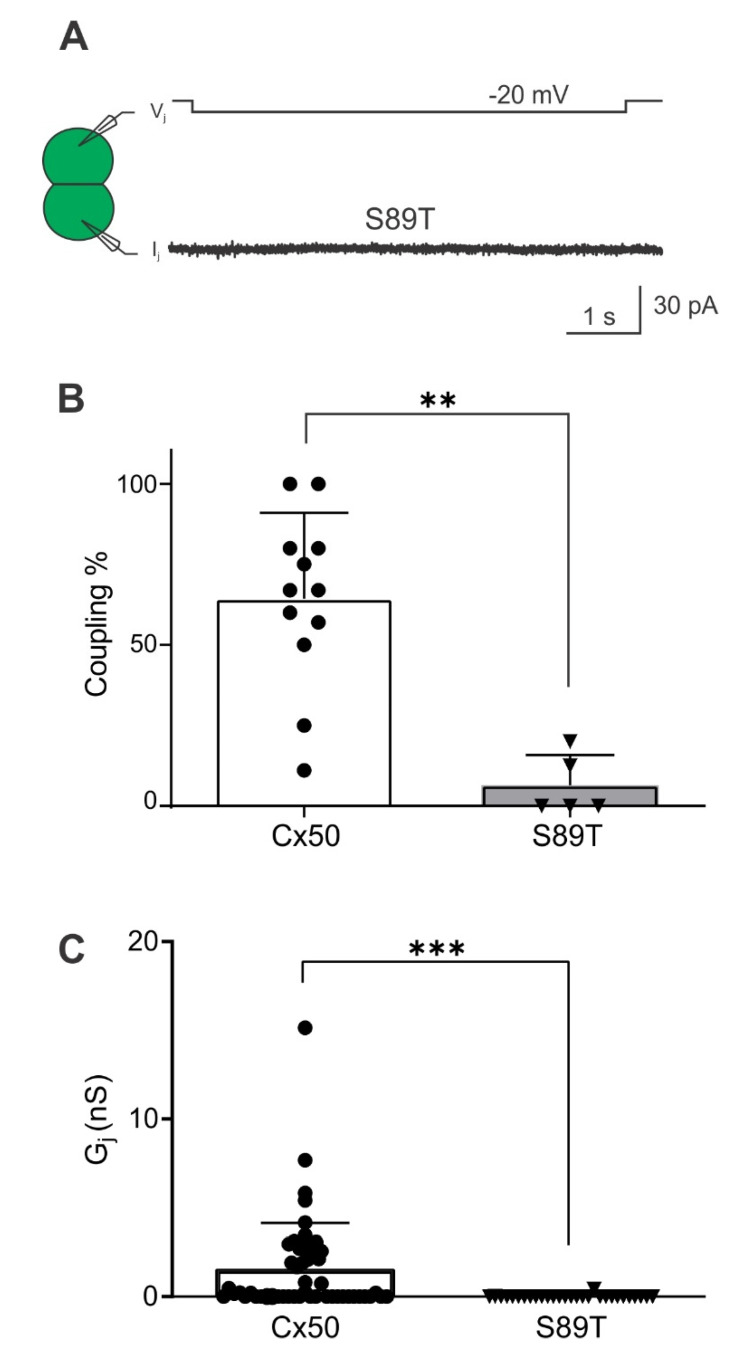
Cx50 S89T failed to form functional GJs. (**A**) A representative I_j_ was recorded from a cell pair expressing Cx50 S89T. No current response was detected with a V_j_ pulse of 20 mV, indicating that S89T failed to form any functional GJs. (**B**,**C**) Summarized bar graphs to show coupling% from five transfections and G_j_ of Cx50 S89T (n = 29 cell pairs), respectively. In both cases, S89T showed a significant reduction in coupling% (** *p* = 0.0011 with Mann–Whitney test) and G_j_ (*** *p* < 0.001 with Mann–Whitney test).

Given these structural predictions, we generated a Cx50 S89T variant construct and expressed this variant in N2A cells for dual patch clamp analysis. Our experimental results are shown in Figure 10. Cx50 S89T failed to form functional GJ channels as both coupling% and G_j_s were significantly reduced to a minimum level compared to wild-type Cx50 GJs. This result and our data on the 14th residue position variants support the structure models, indicating an important role of hydrophobic interactions between the 14th residue position and TM2 residues in Cx46 and Cx50 GJs. Additional experiments are required to further validate these models.

## 3. Discussion

Recently, atomic resolution (1.9 Å) structures on native Cx46 and Cx50 GJs were resolved in an apparent open channel conformation [36], providing excellent templates for structure–function studies on these two GJs. These structure models proposed a novel network of intra- and inter-subunit hydrophobic interactions as the key mechanism anchoring the NT domain to the TM1 and TM2 domains, stabilizing the GJ channel in an open conformation. Our study aimed to examine several individual residues in the NT domain, including hydrophobic residues on the 10th and 14th positions, of these two lens connexins for their roles in GJ function, gating properties, open channel stability, and the single channel conductance (γ_j_). We engineered eight-point variants at the 10th, 13th, 14th, and 15th positions of the NT domain, in which one amino acid residue was exchanged between Cx46 and Cx50. Our results show that all the NT variants were able to form functional GJs in our model cells (N2A), though the Cx46 N13E GJs showed a reduced coupling% and conductance. Conservative exchange of hydrophobic residues, such as L10I and A14V, in Cx46 showed a change in either V_j_-gating or single channel conductance, respectively, indicating that these hydrophobic residues are very important in V_j_-gating and the rate of ion permeation. Looking into the GJ structures of these connexins and homology models of the NT variants, steric clash could be identified on Cx46 L10I and A14V with residues on TM2. Interestingly, mutating one of the A14/V14-interacting residues in the TM2 domain of Cx50, S89T (with a larger side chain), leads to a similar steric clash in the structure model, and, more importantly, this variant failed to form any functional GJs in our model cells. This study, together with our previous study on switching the entire NT domain between these two lens connexins [38], supports the newly proposed model that hydrophobic interactions between the NT and TM2 domain play an important role in stabilizing the NT domain in an open channel conformation. Modifications of these hydrophobic (10th and 14th) as well as hydrophilic residues (9th, 13th, 15th) in the NT domain could lead to changes in coupling%, G_j_, V_j_-gating, unitary channel conductance, and/or open state stability in Cx46, Cx50 (Table S2), and possibly other related connexin GJs.

Sheep connexins were selected to better align our functional data with the resolved high-resolution GJ structure models [18,36]. Sheep and human show 85% overall and 96% structure-resolved domain sequence identity for Cx50, and 66% overall and 95% structure-resolved domain sequence identity for Cx46. Similarly, sheep Cx46 and Cx50 also show high sequence identity with mouse orthologs. More importantly, the NT domains and those residues in the TM2 (89th, 90th, and 93rd residue positions) interacting with the 10th and 14th residues in the NT of Cx46 or Cx50 are 100% identical among sheep, mouse, and human. Thus, we believe that sheep Cx46 and Cx50 are excellent models for the corresponding orthologs from humans, rodents, and possibly other species.

### 3.1. Hydrophobic Residues in the NT Domain of Cx46 and Cx50

Based on the cryo-EM structure models of Cx46 and Cx50 GJs, a large portion of the NT domain (4th–17th residue) forms an α-helix structure with hydrophobic residues facing TM1/2 domains and hydrophilic residues facing the pore. The hydrophobic residues in the NT α-helix showed novel inter- and intra- subunit hydrophobic interactions with hydrophobic or polar residues on TM1/2 domains [18,36], including the 10th and 14th residues in the NT as shown in Figure 9. However, in the Cx26 GJ crystal structure, only a small portion of the NT domain (5th–10th residue) formed an α-helix structure, and the equivalent residues in the Cx26 structure adopted a very different orientation. For example, the hydrophobic residue, I9 of Cx26 (equivalent to 10th residue in Cx46 and Cx50), is directly facing the pore and another hydrophobic residue, V13 (equivalent to 14th residue in Cx46 and Cx50), is at least 4 Å away from any residue on the TM2 domain [29]. In both cases, these residues are unlikely to form any intra-subunit hydrophobic interactions. Molecular dynamic simulations on Cx46 and Cx50 GJ models found that these structures are stable in the proposed open conformation due to the extensive hydrophobic interactions between the NT and TM1/2 domains, unlike that of the Cx26 GJ structure, which is very unstable in the open conformation [18]. It is interesting to note that molecular dynamic simulations on the chimeras of Cx46 and Cx50 with swapped NT domains showed a large increase in NT domain instability in Cx46 50NT GJ (a chimera with Cx50 NT domain replacing that of Cx46) due to steric clash of hydrophobic residues on the NT domain, which correlated with a complete loss of GJ function [38]. These observations indicate an important role of paralogue-specific hydrophobic interactions between NT and TM1/2 in stabilizing the NT domain in open conformation.

Our current study further tested several different residues individually in the NT domains of these two connexins. Consistent with our previous study, we identified that mutation of hydrophobic residues at the 10th and 14th positions in Cx46 could lead to steric clash with residues on the TM2 domain and alter the GJ structure. Surprisingly, single mutant variants were still able to form functional GJs, but with altered V_j_-gating (L10I) or γ_j_ (A14V). In GJs of both L10I and A14V, the open state stability was substantially decreased from that of wild-type Cx46 GJ. Additionally, our designed variant S89T in Cx50 also suggested steric clash between V14 and T89, which resulted in a complete loss of GJ function (Figure 10), demonstrating the importance of the hydrophobic interaction between the NT and TM2 domain for GJ function, V_j_-gating properties, open state stability, and rate of ion permeation. 

Additional lines of evidence support important functional roles of the hydrophobic residues in the NT domain of Cx46 and Cx50. First, several congenital cataract-linked missense mutations occur on these hydrophobic residues, such as L11S in Cx46 as well as W4R and L7P in Cx50 [39,40,41,42,43]. It is interesting to mention that a functional study indicated that L11S failed to form functional GJs [40]. Second, hydrophobic residues are highly conserved in Cx46 and Cx50 as well as among α and other families of connexins, and the equivalent hydrophobic residue positions are almost all able to form amphipathic α-helix to likely interact with their TM1/2 domains. These hydrophobic residue positions are also frequently found to be hot spots for inherited disease-linked connexin mutants, such as oculodentodigital dysplasia-linked mutants in another α-connexin Cx43 (L7, L11) [44] and CMT1X-linked mutants in a β-connexin Cx32 on equivalent positions (W3, L9, L10) [37]. Functional studies on mutations of the hydrophobic NT residues of Cx32, including W3D, L6D, L9D, and L10D all resulted in non-functional GJs [17]. These data collectively emphasize a critical role played by the hydrophobic residues of the NT domain in determining GJ channel function and its properties.

### 3.2. Hydrophilic Residues in the NT Domain of Cx46 and Cx50

In addition to hydrophobic residues determining Cx46 GJ functional properties, Cx46 N13E showed a significantly decreased coupling% as well as macroscopic and unitary channel conductance in the present study. Our homology model on Cx46 N13E showed that this mutation altered the network of intra- and inter-subunit salt-bridge interactions. Some of these interactions, including the R9 with a neighboring subunit E12 salt-bridge, were frequent and were also predicted to exist during molecular dynamic simulation on wild-type Cx46 GJs [38], but a rare frequency intra-subunit salt-bridge interaction between R9 and E13 was observed (Figure 9A). This non-covalent salt-bridge interaction could pull R9 towards the cytosol, destabilizing the open conformation of the NT domain to trigger/facilitate the initial step of GJ closing. A similar interaction may have also played a role in the impairment of V_j_-gating and destabilizing the open state of Cx50 N9R GJ in our previous study [38]. We cannot rule out that other structural changes associated with N13E, including perturbed subunit folding or additional changes in interaction partners, could also play a role in observed reduction in coupling%, G_j_, and γ_j_. 

Reduction of γ_j_ in Cx46 N13E is somewhat surprising, as we predicted that this variant would increase the net negative charge at the GJ pore entrance and lead to enhanced cation concentration locally, thereby increasing the rate of permeation of this cation preferred channel. Previous studies by our group on Cx46 and Cx50 and its variant GJs displayed higher γ_j_ with increasing negative charge of the NT, TM1, and E1 domains (all pore lining domains) [19,28,45] or decreasing positive charge in the NT, such as Cx46 R9N [38]. Apparently, for these two cation-preferring GJ channels, a higher net negative charge in pore-lining residues would be predicted to increase the negativity of pore surface electrostatic potentials, which in general favor increasing local cation concentrations in the pore or the pore entrance area; both, in theory, should increase the rate of permeation or γ_j_. In many of these variants, the γ_j_ was indeed increased [19,28,38,45]. However, caution should be taken, as some additional physical changes are also associated with these charged residue variants. The side chain length (or size) could reduce the pore diameter or establish additional intra- and inter-subunit interactions to alter the permeation passage structure and properties, thereby changing γ_j_ or other gating properties. These additional changes could play a role in our observed reduction in γ_j_ in Cx46 N13E and one of our previously studied Cx50 variants, D3E [25].

Another example of a changed network of intra- and inter-subunit interactions by a missense mutation in a pore-lining residue is the congenital cataract-linked mutation in Cx50, T39R. The T39R Cx50 hemichannels showed partial loss of V_j_-gating, and molecular dynamic simulations showed changes in multiple electrostatic salt-bridge interactions by the introduced R39, including neutralizing the putative voltage-sensing residue D3, as well as a newly established interaction with E42, which could participate in loop gating [46]. Steric factors might also play a role in the reduced coupling conductance of N13E observed in the present study, as glutamic acid has a bulkier side chain than the original asparagine. 

In summary, part of the NT domain formed an α-helix structure with two faces: a hydrophobic face anchors to TM1/2 domains and a hydrophilic face lining the aqueous permeation passage. Both hydrophobic and salt-bridge interactions within or between subunits on NT domain residues are important determinants that shape the V_j_-gating properties, single channel conductance, and channel open stability. More systematic experimental studies on individual missense variants in these lens GJs together with molecular dynamic simulations will help to provide a comprehensive understanding of the V_j_-gating and ion permeation control of these lens and possibly other GJs. Table S2 summarizes the key findings of the present and our previous study on each of the studied variants.

## 4. Materials and Methods

### 4.1. Plasmid Construction

Complementary DNA (cDNA) for both sheep Cx46 (sCx46, also known as Cx44) and Cx50 (sCx50, also known as Cx49) were synthesized and each of them was inserted into an expression vector, pIRES2-EGFP, with an untagged GFP reporter between the restriction enzyme sites, XhoI and EcoRI (NorClone Biotech Laboratories, London, ON, Canada), as previously described [47,48]. The Cx46 and Cx50 variants (Cx46 L10I, N13E, A14V, Q15N and Cx50 I10L, E13N, V14A, N15Q, and S89T) were generated by PCR-mediated site-directed mutagenesis using the corresponding untagged wild-type constructs as templates with the appropriate primers. All wild-type and variant constructs were sequenced to confirm the accuracy of the nucleotide sequences. The primers used to generate these point variants are listed below:

Cx46 L10I1: forward 5′ GC TTC CTG GGG AGA ATC CTA GAG AAC GCC CAG 3′ and reverse 5′ CTG GGC GTT CTC TAG GAT TCT CCC CAG GAA GC 3′

Cx46 N13E: forward 5′ GG AGA CTC CTA GAG GAG GCC CAG GAG CAC TCC 3′ and reverse 5′ GGA GTG CTC CTG GGC CTC CTC TAG GAG TCT CC 3′

Cx46 A14V: forward 5′ CTC CTA GAG AAC GTG CAG GAG CAC TCC 3′ and reverse 5′ GGA GTG CTC CTG CAC GTT CTC TAG GAG 3′

Cx46 Q15N: forward 5′C CTA GAG AAC GCC AAT GAG CAC TCC ACT G 3′ and reverse 5′ C AGT GGA GTG CTC ATT GGC GTT CTC TAG G 3′

Cx50 I10L: forward 5′ GT TTC CTG GGG AAC CTC TTG GAG GAG GTG 3′ and reverse 5′ CAC CTC CTC CAA GAG GTT CCC CAG GAA AC 3′

Cx50 E13N: forward 5′ G GGG AAC ATC TTG GAG AAC GTG AAT GAG CAC 3′ and reverse 5′ GTG CTC ATT CAC GTT CTC CAA GAT GTT CCC C 3′

Cx50 V14A: forward 5′ C ATC TTG GAG GAG GCC AAT GAG CAC TCC ACG 3′ and reverse 5′ CGT GGA GTG CTC ATT GGC CTC CTC CAA GAT G 3′

Cx50 N15Q: forward 5′ C TTG GAG GAG GTG CAG GAG CAC TCC ACG 3′ and reverse 5′ CGT GGA GTG CTC CTG CAC CTC CTC CAA G 3′

Cx50 S89T: forward 5′ GTC TCC ACG CCG ACG CTG GTG TAC GTG 3′ and reverse 5′ CAC GTA CAC CAG CGT CGG CGT GGA GAC 3′

### 4.2. Cell Culture and Transient Transfections

Connexin-deficient mouse neuroblastoma (N2A) cells were used in these studies (American Type Culture Collection, Manassas, VA, USA). On the day before transfection, N2A cells were passaged in 35 mm dishes with Dulbecco’s modified Eagle’s medium (DMEM) containing 10% fetal bovine serum (FBS) (Life Technologies Corporation, Grand Island, NY, USA) to attain around 70% confluence after overnight incubation. The subsequent day, the N2A cells were transfected with 1 μg of each construct using 2 μL of X-tremeGENE HP DNA transfection reagent (Roche Diagnostics GmbH, Indianapolis, IN, USA) in Opti-MEM + GlutaMAX-I medium (Life Technologies Corporation, Grand Island, NY, USA). After 5 h, the medium was changed back to FBS-containing DMEM, and the cells were incubated overnight. On the next day, transfected cells with Cx46 or Cx46 variants were replated onto coverslips for 2 h before transfer to the recording chamber, while N2A cells expressing Cx50 or Cx50 variants were replated for 5 h, each at 50–70% density. Only isolated cell pairs with expression of GFP were selected for dual patch clamp recording.

### 4.3. Electrophysiological Recordings

Glass coverslips with transfected cells were transferred to a recording chamber on a fluorescent microscope (BX51WI, Olympus) using a 40× water immersion lens and FITC or a dual (red and green emission) filter. The chamber was filled with extracellular solution (ECS) (pH 7.4, osmolarity 310–320 mOsm) at room temperature. The ECS contained (in mM): 135 NaCl, 2 CsCl, 2 CaCl_2_, 1 MgCl_2_, 1 BaCl_2_, 10 HEPES, 5 KCl, 5 D-(+)-glucose, 2 sodium pyruvate. The dual whole-cell voltage clamp technique was then used to characterize the GJs in isolated reporter GFP-positive cell pairs. Patching glass micropipettes were pulled using a micropipette puller (PC-10, Narishige International USA Inc., Amityville, NY, USA). These micropipettes were subsequently filled with intracellular solution (ICS) containing (in mM): 130 CsCl, 10 EGTA, 0.5 CaCl_2_, 5 Na_2_ATP, 10 HEPES (pH 7.2, osmolarity 290–300 mOsm). One of the cells was voltage clamped at 0 mV (junctional current recording cell), while a series of voltage pulses ranging from ±20 to ±100 mV (pulsing cell) was administered to the other cell to establish transjunctional voltage (V_j_). If functional GJ channels existed between the cell pairs, a transjunctional current (I_j_) was measured at the recording cell via a patch clamp amplifier (MultiClamp 700A, Molecular Devices, Sunnyvale, CA, USA) with a low-pass filter (cut-off frequency 1 kHz) and digitized at a 10 kHz sampling frequency via an ADDA converter (Digidata 1322A; Molecular Devices, Sunnyvale, CA, USA). Coupling percentage (coupling%) represents the number of cell pairs coupled out of the total number of cell pairs recorded for each transfection (recordings of 3–8 cell pairs).

### 4.4. Transjunctional Voltage Dependent Gating

A voltage-step protocol (±20 to ±100 mV with 20 mV increment) was applied on the “pulsing cell” of the cell pair expressing the construct of interest to establish transjunctional voltage (V_j_). Transjunctional current (I_j_) was then obtained in the recording cell. V_j_-dependent gating (V_j_-gating) reflects the I_j_ amplitude decline with time at high V_j_ (±40–100 mV). Coupling conductance (G_j_) can be calculated via the equation (G_j_ = I_j_/V_j_). Only cell pairs with G_j_ lower than 9 nS were chosen to study the V_j_-gating to minimize the voltage clamp errors [22,49,50,51]. The peak amplitude of junctional current (I_peak_) was measured at the beginning of the I_j_, while the steady-state amplitude of I_j_ was measured by taking the average junctional current of the last 500 ms of each trace. The steady-state G_j_ is normalized to the peak G_j_ to obtain a normalized steady-state junctional conductance (G_j,ss_) which can be plotted with the entire tested V_j_ range. This G_j,ss_–V_j_ relationship can be described by a two-state Boltzmann equation:

$$G_{j,\mathrm{ss}}=\frac{G_{\max}\;–{\;G}_{\min}}{1+\;\exp\left[A\left(V_{j}-V_{0}\right)\right]}+G_{\min}$$

V_0_ is the voltage at which the conductance is reduced by half [(G_max_–G_min_)/2], G_max_ is the maximum normalized conductance, G_min_ is the normalized voltage-insensitive residual conductance, and parameter *A* is the slope of the fitted curve which reflects the V_j_ sensitivity [12,52].

### 4.5. Single Channel Analysis

Unitary channel currents (i_j_s) can be identified in cell pairs with 1–3 GJ channels. The recorded i_j_s were filtered using a low-pass Gaussian filter (200 Hz) in Clampfit10.3 (Molecular Devices, Sunnyvale, CA, USA). All-point histograms were used to determine baseline and open state amplitude for each current after fitting these histograms with Gaussian functions. Average i_j_s at each V_j_ (regardless of V_j_ polarity) of different cell pairs were used to generate the i_j_–V_j_ plot. Slope single channel conductance (γ_j_) was obtained by linear regression of the i_j_–V_j_ plot. Single channel open state dwell time was measured and the average open dwell time was plotted similar as described earlier [38]. 

### 4.6. Homology Structure Modeling

Homology structure models of missense variants were generated based on their respective template using Modeller (version 9.17, Andrej Sali Lab, San Francisco, CA, USA) [53]. For example, the Cx46 variant models were based on the Cx46 GJ cryogenic electron microscopy-derived structure (7JKC) and the Cx50 variants were based on Cx50 GJ cryogenic electron microscopy-derived structure (7JJP) [36]. A total of 10 homology models were created for each variant using the default Modeller settings and in the absence of symmetry restraints. The model showing the lowest discrete optimized energy (DOPE) score was taken as the best model and was visualized. PyMOL (version 2.4.1) was used for the cartoon representations of the backbone structure with variants of interests using stick/sphere views, as needed. Because Modeller minimizes steric clash by introducing small structural rearrangements, the potential for steric clash with surrounding residues was evaluated by directly exchanging the residue on the Cx46 (7JKC) or Cx50 (7JJP) template structures in PyMOL, as indicated.

### 4.7. Statistical Analysis

Data are presented as mean ± standard deviation (SD). Kruskal–Wallis test followed by Dunn’s test were used to compare coupling percentage, G_j_, and γ_j_ of each variant to its corresponding wild-type. Statistical significance is indicated with asterisks (* *p* < 0.05, ** *p* < 0.01, *** *p* < 0.001). Data were analyzed and plotted using GraphPad Prism (GraphPad Software version 9.31, La Jolla, CA, USA).

## 5. Conclusions

In conclusion, our findings indicate that switching the hydrophobic residues at the 10th or 14th positions of Cx46 with those corresponding residues individually in Cx50 can alter V_j_-gating, coupling conductance, γ_j_, and/or open channel stability, while switching these residues individually in Cx50 with those of Cx46 showed relatively little change in V_j_-gating, coupling conductance, γ_j_, and open channel stability. Our results and the homology models are consistent with a previously proposed structure model where hydrophobic residues at the 10th and 14th positions in the NT domain form hydrophobic interactions with residues in the TM2 domain to anchor/stabilize the NT domain in open conformation. In addition, switching the 13th residues between Cx46 and Cx50 could promote novel intra- and inter-subunit interactions to alter coupling%, coupling conductance, γ_j_, and open stability. 

## Figures and Tables

**Table 1:** Boltzmann fitting parameters for the GJs of Cx46 and its variants.

GJs	V_j_ Polarity	G_min_	V_0_ (mV)	*A*
Cx46	+	0.24 ± 0.07 ^1^	39.1 ± 5.5	0.09 ± 0.04
(n = 5)	−	0.23 ± 0.09	38.6 ± 6.8	0.11 ± 0.09
L10I	+	0.22 ± 0.30	56.1 ± 17.8	0.06 ± 0.06
(n = 4)	−	N/A	N/A	N/A
N13E	+	0.17 ± 0.04	33.6 ± 3.6	0.16 ± 0.06
(n = 4)	−	0.17 ± 0.04	32.9 ± 1.8	0.15 ± 0.06
A14V	+	0.24 ± 0.04	35.1 ± 4.2	0.19 ± 0.14
(n = 4)	−	0.15 ± 0.04	37.1 ± 3.0	0.14 ± 0.08
Q15N	+	0.27 ± 0.12	37.3 ± 12.4	0.06 ± 0.04
(n = 4)	−	0.25 ± 0.08	32.3 ± 7.8	0.09 ± 0.06

^1^ Data are presented as mean ± SD and V_0_ are absolute values. Student’s *t*-test was used to compare the Boltzmann fitting parameters of each variant against those of wild-type at the same V_j_ polarity. No parameter showed statistically significant differences between each variant and wild-type GJs. N/A, not applicable.

**Table 2:** Boltzmann fitting parameters for the GJs of Cx50 and its variants.

GJs	V_j_ Polarity	G_min_	V_0_ (mV)	*A*
Cx50	+	0.20 ± 0.15 ^1^	41.5 ± 11.8	0.07 ± 0.05
(n = 6)	−	0.25 ± 0.15	43.6 ± 11.8	0.07 ± 0.05
I10L	+	0.22 ± 0.04	33.0 ± 5.0	0.11 ± 0.04
(n = 4)	−	0.22 ± 0.06	33.7 ± 4.2	0.17 ± 0.10
E13N	+	0.16 ± 0.12	33.2 ± 6.9	0.1 ± 0.17
(n = 3)	−	0.29 ± 0.14	30.0 ± 11.7	0.1 ± 0.14
V14A	+	0.23 ± 0.08	45.4 ± 6.8	0.13 ± 0.10
(n = 4)	−	0.23 ± 0.08	37.8 ± 5.8	0.14 ± 0.14
N15Q	+	0.23 ± 0.13	37.1 ± 12.5	0.08 ± 0.07
(n = 5)	−	0.18 ± 0.11	30.6 ± 11.7	0.09 ± 0.09

^1^ Data are presented as mean ± SD and V_0_ are absolute values. Student’s *t*-test was used to compare the Boltzmann fitting parameters of each variant against those of wild-type at the same V_j_ polarity. No parameter showed statistically significant differences between each variant and wild-type GJs.

## Data Availability

Data in our manuscript are available on request.

## Supplementary Materials

The following supporting information can be downloaded at: https://www.mdpi.com/article/10.3390/ijms231911605/s1.
