# Supplementary material for: The Hydrophobic Residues in Amino Terminal Domains of Cx46 and Cx50 Are Important for Their Gap Junction Channel Ion Permeation and Gating
Source: Int J Mol Sci. 2022 Oct 1;23(19):11605. doi: 10.3390/ijms231911605 (PMC9570504; doi:10.3390/ijms231911605)
Supplement: Supplementary file 1 [file ijms-23-11605-s001.zip › ijms-1929959-supplementary.pdf]

Supplementary Materials

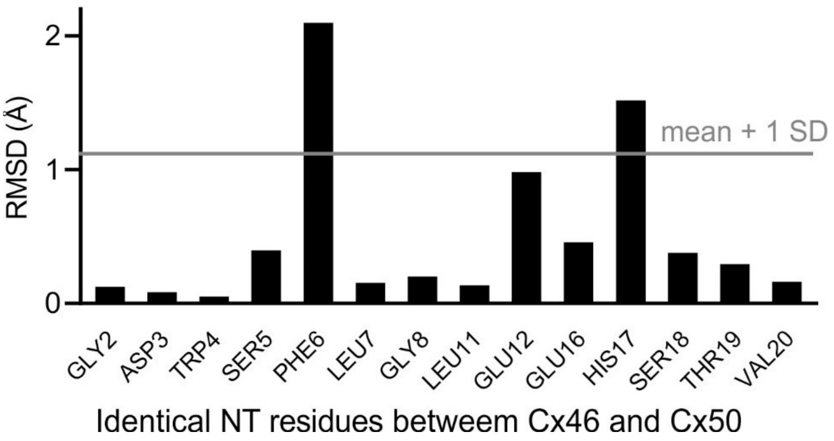

Figure S1. All atom root mean square deviation (RMSD) plot showing differences in the conformations of identical residues in the Cx46 and Cx50 NT domain. The Cx46 (7JKC) and Cx50 (7JJP) GJ structures were backbone atom aligned, excluding the NT domain (2-20 residues). 14 / 19 of the structure-resolved NT residues are identical between Cx46 and Cx50, but the alignment reveals quite different side-chain orientations, such as for Phe6 and His17, which show all atom RMSDs > mean + 1 SD. These differences imply that the packing of those non-identical NT residues between Cx46 and Cx50 could play a role in these structural and functional variations in the NT domain.

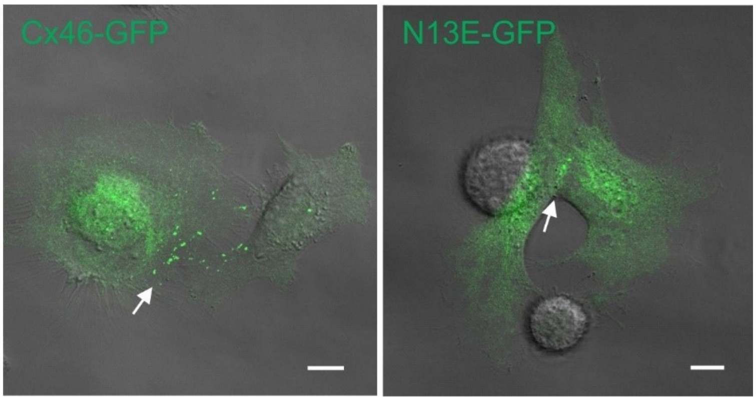

Figure S2. Cx46 N13E-GFP was able to reach cell-cell junctions to form gap junction plaque-like structures. Fluorescent photographs showing that GFP-tagged Cx46 N13E (N13E-GFP) was able to reach cell-cell junctions to form GJ plaque-like structures (as indicated by arrow) similar to that of Cx46-GFP. GFP tag was linked to Cx46 N13E or wildtype Cx46 at the carboxyl terminus.

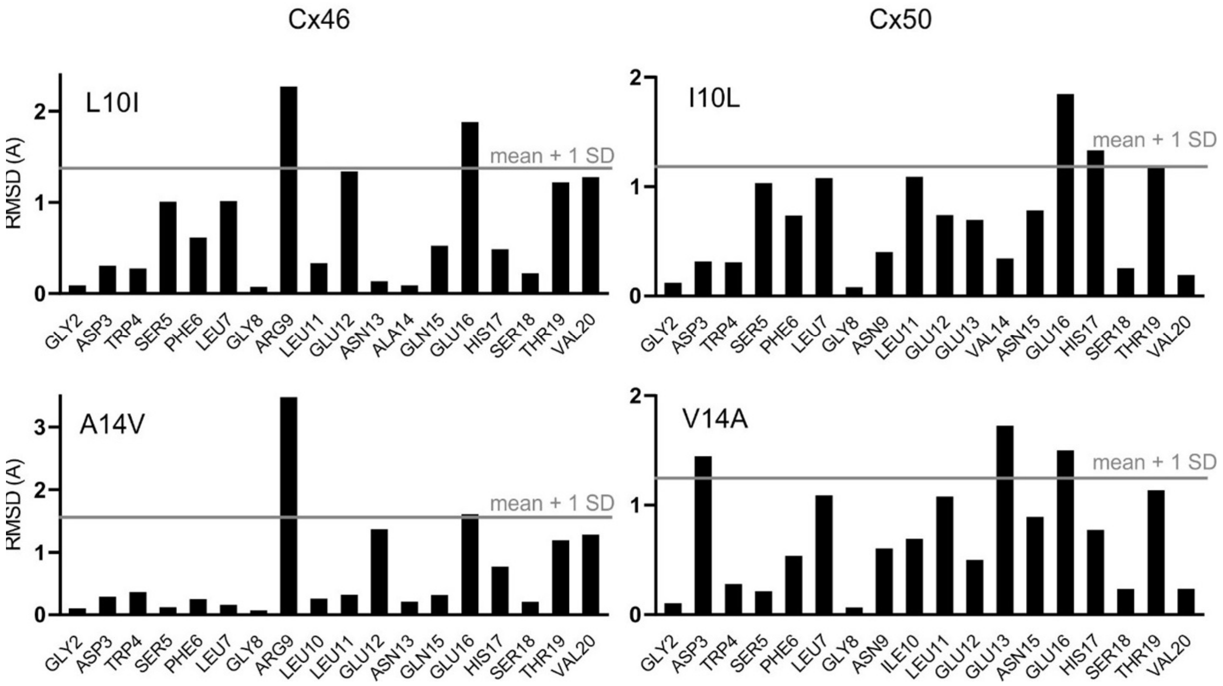

Figure S3. All atom root mean square deviation (RMSD) plot showing differences in the NT domain side chain conformations of homology modeled Cx46 and Cx50 variants. Each of the variant structure models was generated with Modeller using the wildtype Cx46 (7JKC) or Cx50 (7JJP) GJ structures as templates. The generated models were backbone atom aligned to the wildtype Cx46 and Cx50 structures using residues 21-224 or 21-236, respectively (i.e. excluding the NT domain). The all atom RMSD is shown for each identical residue through the NT domain after the alignment, and the mean RMSD + 1 SD of these NT domain residues is indicated with a grey line in each plot. These calculations reveal differences in residue orientations for several NT residues caused by each variant, which could play a role in the observed changes in GJ function.

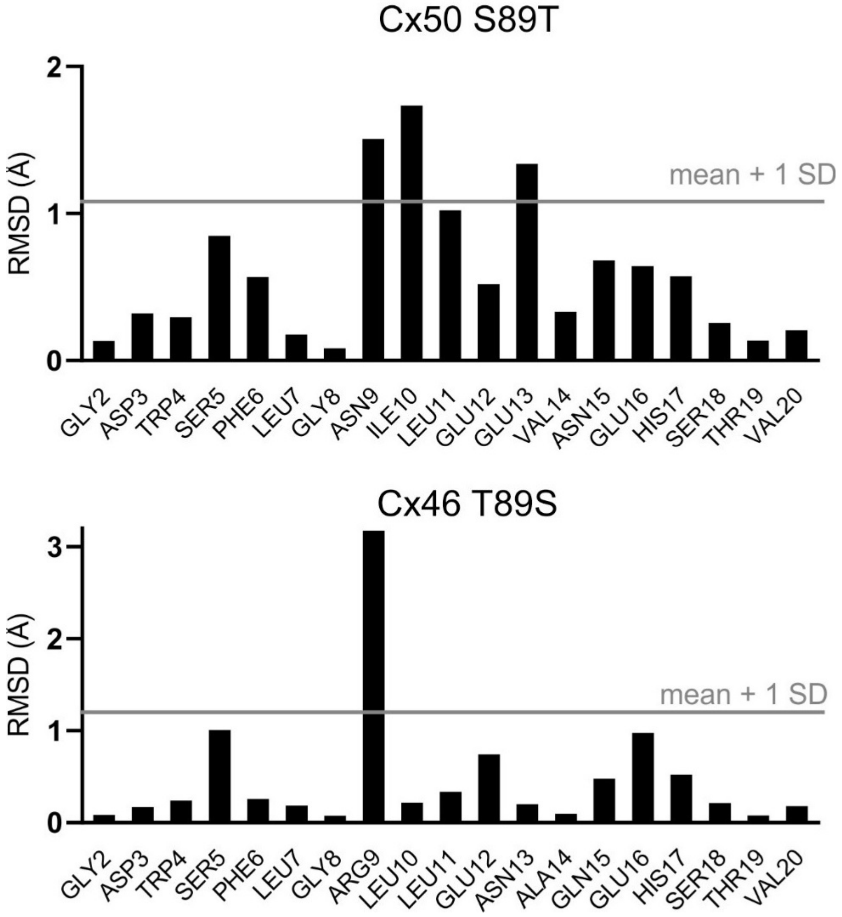

Figure S4. All atom root mean square deviation (RMSD) plots showing differences in the NT domain side chain conformations of homology modeled Cx50 S89T and Cx46 T89S variants. Each of these variant structure models was generated with Modeller using the wildtype Cx46 (7JKC) and Cx50 (7JJP) GJ structures as templates. The generated models were backbone atom aligned to the wildtype Cx46 and Cx50 structures using residues 21-224 or 21-236, respectively (i.e. excluding the NT domain). The all atom RMSD is shown for each identical residue through the NT domain after the alignment, and the mean RMSD + 1 SD of these NT domain residues is indicated with a grey line in each plot. These calculations reveal Cx50 S89T showed more NT residues with large changes in their positions compared to Cx46 T89S, consistent with the notion that the T89S substitution is more accommodating.

Table S1. Gap junction channel open dwell times for Cx46 and Cx50 variants.

Vjs	40 mV					60 mV					80 mV					100 mV				
Variant	Mean (ms)	SD	Cell pairs	N	P	Mean (ms)	SD	Cell pairs	N	P	Mean (ms)	SD	Cell pairs	N	P	Mean (ms)	SD	Cell pairs	N	P
**Cx50 WT**	114.5	174.1	3	130		94.6	140.8	3	96		70.1	74.4	3	63		39.8	63.5	3	49	
**Cx50 I10L**	132.6	199.8	7	314		191.2	293.4	6	43	**	44.7	221.0	7	267	***	92.4	166.4	7	45	
**Cx50 E13N**	60.5	105.4	6	166	***	108.8	456.1	6	77		40.6	73.8	2	49	**	27.1	42.6	5	170	
**Cx50 V14A**	121.9	255.2	8	250		122.4	304.1	9	236		203.2	235.7	7	42	*	204.5	262.5	5	40	***
**Cx50 N15Q**	69.1	132.5	6	216	**	46.4	131.4	5	315	**	47.6	73.1	5	76	*	33.3	48.3	5	61	
**Cx46 WT**	285.8	404.6	7	75		228.2	251.2	7	61		110.7	164.0	7	65		36.6	47.4	7	214	
**Cx46 L10I**	10.1	21.1	4	289	***	12.1	48.9	6	265	***	8.9	15.9	6	258	***	9.0	16.2	6	349	***
**Cx46 N13E**	15.1	34.7	5	474	***	61.6	102.0	5	80	***	25.4	44.6	4	83	***	15.5	20.2	4	25	*
**Cx46 A14V**	77.0	104.3	3	195	***	97.1	155.2	2	47	***	53.4	74.8	6	95	**	24.5	36.4	4	306	***
**Cx46 Q15N**	65.5	178.4	5	157	***	17.7	56.1	5	637	***	10.8	21.0	4	500	***	11.8	18.9	3	35	***

Mean open dwell time (in ms), standard deviation (SD), number of cell pairs, number of transfections (N), and statistical difference (P) are shown for each variant and wildtype Cx46 and Cx50. Mann Whitney test was used to assess the statistical difference between dwell times of each mutant vs their wildtype at each tested Vj. Blank represents no difference; * $P < 0.05$; **$P < 0.01$; ***$P < 0.001$

Table S2. Summary of functional changes observed in each variant GJ as compared to corresponding wildtype Cx46 or Cx50 GJ.

Connexin	Variant	Functional changes relative to their respective wildtype GJs
Cx46	50NT*	○ Non-functional GJs<br>○ Steric clash with TM2 residues
	R9N*	○ Increased γ_j<br>○ Shorter open-state dwell time
	L10I	○ Loss of Vj gating on one polarity<br>○ Shorter open-state dwell time<br>○ Steric clash with L90
	N13E	○ Decreased coupling %<br>○ Decreased G_j and γ_j<br>○ Shorter open-state dwell time<br>○ Occasional salt bridge interaction with R9
	A14V	○ Decreased G_j and γ_j<br>○ Shorter open-state dwell time<br>○ Steric clash with T89
	Q15N	○ Shorter Open-state dwell time
Cx50	46NT*	○ Decreased γ_j<br>○ Impaired V_j-gating<br>○ Longer open-state dwell time
	N9R*	○ Decreased γ_j<br>○ Loss of V_j gating<br>○ Shorter open-state dwell time
	I10L	○ Open-state dwell time could be longer or shorter at some V_js
	E13N	○ Decreased G_j and γ_j<br>○ Shorter open-state dwell time some V_js<br>○ Decreased net negative charge in the NT domain
	V14A	○ Longer open-State dwell time at high V_js (80 and 100 mV)
	N15Q	○ Decreased G_j and γ_j<br>○ Shorter open-state dwell time at some tested V_js
	S89T	○ Non-functional GJs<br>○ Steric clash with V14

G_j, Coupling conductance; γ_j, single channel conductance.

*Data from Yue et al. 2021 (J. Physiol. 599, 3313 - 3335).
